# Supplementary material for: Left ventricular segmentation method based on optimized UNet and improved CBAM: ESV and EDV tracking study
Source: PLoS One. 2025 Jun 25;20(6):e0325794. doi: 10.1371/journal.pone.0325794 (PMC12193590; doi:10.1371/journal.pone.0325794)
Supplement: S1 File — (ZIP) [file pone.0325794.s001.zip › heart/a4c-video-dir/describe.docx]

The dataset needs to be obtained from Stanford University School of Medicine
