# Supplementary material for: Left ventricular segmentation method based on optimized UNet and improved CBAM: ESV and EDV tracking study
Source: PLoS One. 2025 Jun 25;20(6):e0325794. doi: 10.1371/journal.pone.0325794 (PMC12193590; doi:10.1371/journal.pone.0325794)
Supplement: Attachment — Submitted filename: Response to Reviewers.docx [file pone.0325794.s004.docx]

**Dear reviewer,**

**We sincerely thank the editor and all reviewers for their valuable feedback that we have used to improve the quality of our manuscript. The reviewer comments are laid out below in italicized font and specific concern shave been numbered. Our response is given in normal font and changes/additions to the manuscript are given in Highlight Text.**

***Reviewer 1***

***1.Simplify overly complex sentences. For example, instead of "These need to be refined for clarity and improved flow," consider "These should be clearer and easier to read." This avoids unnecessary complication while maintaining meaning. Correct grammatical errors to ensure smooth, intuitive reading. Alignment is not good. Avoid the usage of We / I in the article. While using the Acronyms first time, expand them. For example CBAM in abstract.***

**Response:** Thank you for your thorough review of our paper and for providing many valuable comments. We highly value your feedback and have diligently addressed the issues you raised during the revision process. In response to the specific points you identified, we have taken the following measures:

1. **Simplified Complex Sentences**: We have simplified the complex sentences in the manuscript to ensure that the expression is clearer and more concise. We have avoided lengthy and hard-to-understand sentence structures, ensuring that each sentence is succinct and can smoothly convey the research content.
2. **Corrected Grammatical Errors**: We have conducted a comprehensive grammar check of the entire manuscript to ensure that no grammatical errors were overlooked. To improve the language quality, we used professional grammar checking tools and also performed manual proofreading to ensure that every sentence adheres to grammatical rules.
3. **Improved Readability and Intuitiveness**: In the process of simplifying sentences, we paid special attention to the logical structure of the manuscript and the fluency of the language to ensure that the overall expression is natural and smooth, avoiding confusion for readers. Additionally, we ensured smoother transitions between different sections to enhance the readability of the paper.
4. **Adjusted Alignment Issues**: We have carefully reviewed the formatting of the entire manuscript to ensure that paragraphs, titles, tables, and figures are aligned according to the journal's requirements. We have addressed any non-standard alignment issues to improve the overall layout of the paper.
5. **Avoided Using "We/I"**: We have noted that avoiding the use of the first person is a common norm in academic writing. Therefore, we adjusted the relevant sections of the manuscript to use more objective expressions, avoiding the use of "We" or "I," and ensuring a more neutral and formal tone.
6. **Expanded Explanations of Technical Terms**: We have provided detailed explanations for technical terms the first time they appear in the manuscript, such as CBAM, UNet, SimAM, etc., to ensure that readers have a clearer understanding of the content.

We have comprehensively revised the manuscript based on your feedback and ensured that these issues have been properly addressed. Once again, thank you for your careful review of our paper and your valuable suggestions. We have completed these revisions and submitted the revised version, looking forward to your further review.

If you have any other suggestions or requests, we are very happy to continue making improvements.

***2. For mechanisms like CBAM and SimAM, providing more context is key. Explain them in simpler terms, offering real-world analogies if possible. This will help a wider audience grasp the technical aspects without needing a specialized background.***

**Response:** Thank you for your valuable suggestions regarding our paper. We fully understand the importance of providing more context and using simpler terminology to clearly explain our research content. To enhance the readability and comprehensibility of the paper, we have implemented the following improvements:

1. **Using Simpler Terminology**: We have reviewed the manuscript for any overly technical or obscure terms and have replaced them with simpler, more easily understandable language to ensure that the paper is accessible to a broad readership. For instance, we have explained some highly specialized terms to prevent any confusion among readers.
2. **Providing Real-World Analogies**: We have attempted to clarify some abstract or complex concepts by using real-world analogies. By drawing parallels between our theoretical or experimental results and everyday experiences, we aim to make the research content more intuitive and easier to grasp. For example, when explaining CBAM, we have likened it to observing a busy street filled with people, vehicles, and buildings. If you are looking for a friend, your brain naturally focuses on the crowd, ignoring irrelevant elements like parked cars or buildings.
3. **Simplifying Expression**: In addition to supplementing terminology and context, we have simplified certain sentence structures within the manuscript to make the expression more direct and clear. This involves avoiding overly complex sentence constructions to ensure the paper flows more smoothly and is easier to read.

We believe these improvements have contributed to enhancing the paper's readability and comprehensibility, ensuring that readers can effortlessly understand the research content and conclusions with minimal confusion. Once again, thank you for your invaluable suggestions. Your feedback has been instrumental in helping us improve the quality of our paper. We have promptly completed these modifications and submitted the revised version, looking forward to your further review.

If you have any additional suggestions or requests, please feel free to let us know, and we will continue to make improvements.

**P8**

Convolutional Block Attention Module (CBAM) is an attention mechanism for convolutional neural networks, which combines channel attention and spatial attention to enhance the representation ability of feature maps. CBAM improves the performance of the model by adaptively weighting the input feature map to highlight the important features and suppress irrelevant ones. CBAM consists of two sub-modules: the Channel Attention Module (CAM) and the Spatial Attention Module (SAM), as shown in Fig 3. CBAM is similar to how our brains focus attention on important parts when observing something. Imagine you are looking at a busy street filled with people, vehicles, and buildings. If you are searching for a friend, your brain naturally concentrates on the crowd, ignoring irrelevant elements like parked cars or buildings. This is exactly what CBAM does in neural networks—it helps the model focus on "important" features, such as distinguishing people from vehicles in an image.

**P10**

The SimAM (Simple, Parameter-Free Attention Module) [9] design concept is derived from neuroscience theory, aiming to enhance which aims to enhance the representation ability of convolutional neural networks while remaining lightweight without adding additional parameters. SimAM solves two major problems with existing attention modules: attention weights are limited to either the channel or spatial dimensions, and there is a lack of flexibility, requiring additional parameters that increase model complexity. Imagine being in a classroom where a teacher is explaining a topic. Some students (features) are more focused because they have studied more and understood the material more deeply. SimAM is like a teacher who can recognize which students are more attentive and concentrate more attention on those students, thereby enhancing the overall learning effectiveness. It gives more attention to students who have a deeper understanding (important features) while ignoring those who are less engaged (irrelevant features).

***3.The addition of attention mechanisms may slow things down, especially in real-time applications. Include a discussion on how to tweak or optimize the model so it remains effective for real-time use without losing too much of its complexity. This section should speak directly to practical challenges and solutions.***

**Response:** Thank you for your valuable suggestions on our paper. Based on your feedback, we have refined the paper to focus more on the practical challenges and our proposed solutions, thereby enhancing its relevance and practicality.

To avoid a significant reduction in inference speed when incorporating attention mechanisms (SCBAM), we have adopted the following optimization strategies:

1. In the PrunedNestedUNet structure, we utilized a nested U-Net (DA-UNet++), a variant of U-Net designed to extract features at multiple scales through several convolutional blocks and upsampling operations. By reducing the number of convolutional layers or channels within the layers, we further minimized computational overhead. Specifically, we pruned redundant layers or channels in the U-Net, removing unnecessary convolutional kernels and neurons. This approach reduced the model size and computational complexity, thereby improving inference speed.
2. When applying attention mechanisms, we reduced computational costs by adjusting the kernel size of the attention module. For example, in the spatial attention module of CBAM (Convolutional Block Attention Module), we reduced the kernel size of the convolution operation, alleviating computational burden while still retaining a reasonable level of attention effectiveness.

We believe these improvements have significantly enhanced the readability and comprehensibility of the paper, ensuring that readers can intuitively understand the core content and solutions of our research. Once again, we sincerely appreciate your valuable feedback, which has been instrumental in improving the quality of our paper. The revised version has been submitted promptly, and we look forward to your further review.

If you have any additional suggestions or requirements, please do not hesitate to inform us. We will continue to make improvements.

**P8**

By combining these attention mechanisms, the model can more effectively focus on key regions and important channels in the image, thereby improving segmentation accuracy. To prevent the SCBAM attention mechanism from significantly reducing the model's inference speed, this study employs the following optimization methods:

- Pruned Nested U-Net Structure: By reducing the number of convolutional layers and pruning redundant convolutional kernels and neurons, the computational overhead is decreased, the model size is reduced, and inference speed is enhanced.
- Adjusting the Kernel Size of the Attention Mechanism: When using the spatial attention module of CBAM, smaller convolutional kernels are employed to reduce the computational burden while maintaining effective attention mechanisms.

***4.The EchoNet-Dynamic dataset is central to the paper, but expanding the discussion to how the model might work with other datasets—specifically in medical contexts—will make the findings more impactful. A brief note on cross-validation or potential limitations would be helpful here***

**Response:** Thank you for your valuable suggestions on our paper. Based on your feedback, we have further expanded the discussion on the model’s applications in the medical field, particularly focusing on how it can collaborate with other datasets to enhance the impact of the research results.

First, although the EchoNet-Dynamic dataset has provided a solid foundation for training and evaluating the model, we recognize that the model’s performance on other medical datasets is equally important, especially in the medical field where the model’s generalization ability is crucial. The pruned nested U-Net architecture used in this study, combined with SCBAM (Convolutional Block Attention Module), has demonstrated good versatility, making it suitable for other medical image segmentation tasks that require fine-grained feature extraction and attention mechanisms.

However, despite the excellent performance of the model on the EchoNet-Dynamic dataset, we also realize that there may be potential limitations when applying the model to new datasets or other medical imaging tasks. For example, many medical datasets, especially those from specialized fields such as echocardiography, may be limited in terms of scale and quality, with lower resolution or incomplete annotations, which could affect the model’s performance. Furthermore, models trained specifically for one type of medical imaging data may not transfer directly to other domains. For instance, X-ray or MRI images differ significantly from cardiac ultrasound images in contrast, noise, and resolution, which may present challenges when trying to transfer the model.

Additionally, while attention-based models have performed well in various tasks, their interpretability remains a critical issue in the medical field. Clinical decision-making often relies on a high level of trust and transparency in the model’s outputs, making the improvement of model interpretability essential for its broad application.

To address these challenges, we will continue to conduct in-depth research to explore solutions and improve the model’s applicability and reliability across different medical applications. We appreciate your invaluable feedback and look forward to your further review of the revised version.

If you have any additional questions or requests, please feel free to let us know, and we will continue to make improvements.

**P17**

While the EchoNet-Dynamic dataset provides a robust foundation for model training and evaluation, generalization to other medical datasets remains a key concern The Pruned Nested U-Net with SCBAM demonstrates versatility, making it suitable for various medical image segmentation tasks requiring fine feature extraction and attention mechanisms. However, challenges remain when applying the model to new datasets. Many medical datasets, especially in specialized fields like echocardiography, are limited in size, resolution, or annotation quality. Additionally, models trained on one type of imaging, such as cardiac ultrasound, may not transfer well to others like X-rays or MRIs due to differences in contrast, noise, and resolution. Furthermore, while attention-based models excel in performance, their interpretability still needs improvement, a key requirement for clinical applications. Future work will focus on addressing these limitations to enhance the model's generalizability and reliability in diverse medical contexts.

***5.What are all the features extracted? How many are used for segmentation? What is the reason behind the selection of features?***

**Response:** Thank you for your valuable suggestions. Based on your feedback, we will explicitly explain the reasons for choosing the Nested U-Net architecture, CBAM (Convolutional Block Attention Module), and SimAM (Similarity Attention Module) in our paper, and provide a detailed discussion on their application value in our research.

Feature extraction is primarily performed through the Nested U-Net architecture, combined with the attention mechanisms SCBAM (CBAM and SimAM). These features are used to guide the segmentation process with the goal of helping the model focus on the most important regions of the image (the regions that need segmentation). Below is an overview of feature extraction and its use in segmentation:

1. **Feature Extraction in the Model Architecture**
   - The Nested U-Net architecture extracts hierarchical features through convolutional layers at different depths, capturing both local and global spatial information in the image.
   - CBAM extracts channel attention features and spatial attention features. Channel attention focuses on the most important channels in the feature map, enhancing useful feature channels. Spatial attention focuses on specific regions in the image (spatial dimension), enhancing spatial features.
   - SimAM (Self-Information Adaptive Module) extracts adaptive features by focusing on the variance in the feature map. It computes an "energy function" to independently adjust the attention of each feature map.

These components collectively ensure that the model can focus on important channels and specific spatial regions in the image, thus improving segmentation performance.

2. **Features Used for Segmentation**
   - The features extracted by the Nested U-Net architecture are processed and used for the segmentation task. Specifically, the hierarchical features in the nested structure correspond to different levels of abstraction, from low-level details (edges, textures) to high-level semantic information (objects, regions to be segmented).
   - CBAM is used to enhance the features by adjusting their importance based on the channel and spatial attention mechanisms. Similarly, SimAM adjusts the focus based on the variance in the feature map. The final segmentation result is obtained by mapping the previous feature maps to the desired output channels through a 1x1 convolution layer.
3. **Reasons for Feature Selection:**
   - **Channel Attention:** Channel attention allows the model to focus on the most important features for the task (such as the correct information labeled by experts). By assigning higher weights to more important channels, the model can suppress irrelevant features.
   - **Spatial Attention:** Spatial attention helps the model concentrate on the most important regions in the image for segmentation. This is particularly useful when only specific parts of the image need to be segmented (such as organs in medical imaging).
   - **SimAM:** By adjusting based on the variance in the feature map, SimAM ensures the model focuses on areas with higher variance, which are typically associated with edges or transitions between different structures.

The primary reason for selecting these features is that the attention mechanisms (SCBAM and SimAM) allow the model to focus on the most relevant parts of the image, thus improving segmentation accuracy. These mechanisms help the model enhance its attention to key regions and channels, leading to better segmentation performance.

4. **Final Feature Representation for Segmentation:**
   - After feature extraction via the Nested U-Net architecture and refinement through attention mechanisms, the features are passed through a 1x1 convolution layer to generate the segmentation map, which represents the probability of each pixel belonging to a specific class (for binary segmentation tasks).

By discussing in detail the reasons for choosing the Nested U-Net architecture and SCBAM, we aim to clearly convey our thought process in model design to the readers, explaining why these features effectively address key challenges in medical image analysis and enhance the overall performance of the model.

**P7**

On this basis, an improved nested UNet structure (NestedUNet) is proposed. Through a nested design, it enhances segmentation performance, the network is able to capture more expressive features. First, 1×1 convolution is performed on the input to adjust the number of channels and preserve the original input information. Then, the spatial dimension of the input is halved by the maximum pooling layer, and the feature map is fed into the first VGG module for two convolution operations. Then, the spatial dimension of the feature map was doubled by transposing the convolution for upsampling, and the upsampled feature map is concatenated with the previously saved map in the channel dimension, and the convolution operation was performed twice in the second VGG module again. At the same time, the SCBAM module is introduced for optimization. Nested U-Net captures richer contextual information by fusing feature maps at different scales through multi-level pooling and upsampling operations. This multi-scale feature fusion strategy significantly improves the model's performance in segmentation tasks, especially in object edge recognition and complex detail handling. Meanwhile, the Nested U-Net architecture extracts hierarchical features through convolutional layers at different depths, capturing both local spatial information and preserving global spatial information. These features are extracted layer by layer, from low-level details (such as edges and textures) to high-level semantic information (such as objects and segmented regions), reflecting different levels of abstraction and further enhancing segmentation accuracy. In addition, the introduction of the VGG module enables the model to extract and retain more low-level edge information, reduce the ambiguous area in the segmentation result, and further improve the accuracy.

**P8**

CBAM enhances feature representation by extracting both channel and spatial attention features. The channel attention mechanism focuses on the most important channels, improving the representation of key feature channels. The spatial attention mechanism, on the other hand, focuses on specific regions of the image, thereby enhancing spatial features. The channel attention mechanism helps the model concentrate on the most relevant features for the task, while spatial attention emphasizes critical areas of the image.

SimAM, in contrast, emphasizes the local information of each pixel in the feature map. It highlights important features while reducing noise interference through a parameter-free energy function calculation. This approach avoids unnecessary global computations, thus improving computational efficiency. SimAM adjusts the attention of each feature map by calculating its variance, enabling the model to focus adaptively on areas with higher variance, typically associated with edges or transitions between different structures. These mechanisms help the model focus on more meaningful information at various levels, improving segmentation accuracy.

***6.* *While you’ve included numbers and metrics, visuals can tell a different part of the story. Show side-by-side comparisons of segmentation results from different models. These visual cues will help readers quickly see the strengths of your proposed method****.*

**Response:** Thank you for your valuable suggestions. Based on your feedback, we will present the segmentation results with clear and visually appealing boundary comparisons to help readers quickly identify the differences and advantages of the various models.

This study uses boundary line comparison to visually demonstrate the performance differences of different models in segmentation tasks. By drawing boundary lines of the segmentation results and comparing them with the ground truth boundaries, we can highlight the accuracy and refinement of boundary detection by the models.

We use edge detection algorithms to extract the boundaries of both the ground truth and predicted segmentation results. The ground truth mask is correctly loaded and obtained according to the dataset structure. Edge detection is implemented to assist readers in quickly seeing the advantages of our proposed method. The boundary lines of different models should be distinctly distinguishable by using different colors and line styles to avoid confusion. Red boundaries represent the ground truth edges, while green boundaries represent the predicted edges of the current model. These boundaries are overlaid on the original image for comparison. By displaying the real boundaries alongside the boundaries of each model, readers can easily and intuitively compare the results.

In this way, we can effectively demonstrate the advantages of our model in medical image analysis, helping readers understand the effectiveness of the proposed method.

We appreciate your valuable suggestions and look forward to your further review of the revised version.

If you have any additional questions or requests, please feel free to let us know. We will continue to make improvements.

***Reviewer 2***

***1.Figures and Tables are given and explained clearly.***

**Response:** Thank you for your valuable feedback on the figures and tables in our paper. We greatly appreciate your comments and have carefully reviewed the relevant sections. Indeed, in the initial draft, we may not have fully explained the details of the figures and tables, which led to a lack of clarity. To address this, we have made detailed revisions to the titles and captions of the figures and tables. Specifically, we have revised the titles of each figure to ensure that they more accurately reflect the content and purpose of the figure. Additionally, we have provided more detailed explanations for the legends to ensure that the meaning of each symbol, color, and line is clearly explained.

Table explanations: For the tables, we have added more detailed captions, explaining the meaning of each row and column. We have also included clarifications regarding the data sources and outlined the relationships and significance of the various data points, so readers can better understand the content of the tables. Relationship between figures/tables and the main text: We have strengthened the connection between the figures and tables and the main text, ensuring that each figure and table is closely related to the corresponding discussion in the paper, with clear guidance in the text on how to interpret these figures to avoid any misunderstandings by the readers.

Through these revisions, we aim to make the figures and tables more self-explanatory, helping readers easily understand the research findings. We have incorporated these changes in the revised version and look forward to your further review.

Once again, thank you for your valuable feedback, which has helped improve the clarity and quality of our paper. If you have any further suggestions, we would be happy to receive them and make any necessary adjustments.

***2.* *Many grammatical mistakes are there***

**Response:** Thank you for your careful review of our paper and for pointing out the grammatical issues. We highly value your feedback and deeply apologize for any inconvenience this may have caused. We have carefully reviewed the paper and acknowledged the grammatical issues. To improve the language quality, we have taken the following measures:

• **Comprehensive grammar check**: We have used professional grammar-checking tools to conduct a thorough grammar check of the entire paper, ensuring that sentence structure and word usage meet standards, and avoiding grammatical errors and awkward expressions.

• **Paragraph-by-paragraph review**: We have reviewed each section of the paper to ensure logical clarity and accuracy in expression. In particular, we have simplified and restructured some complex sentences to enhance the fluency and readability of the language.

• **Seeking help from native colleagues**: To ensure the language is more natural, we have sought the assistance of native English-speaking colleagues for professional language polishing, ensuring that no grammatical or expression issues are overlooked.

We believe that, after these revisions, the language quality of the paper has significantly improved. Once again, we thank you for pointing out these issues, as they have been very helpful in improving the paper's quality. If you have any further suggestions or areas you would like us to particularly focus on, please feel free to let us know. We look forward to your review of the revised version and sincerely appreciate your patience and support.

***3.* The author have not shown any interest in formatting the document**

**Response:** Thank you for reviewing our paper and pointing out the formatting issues. We highly value the feedback you provided and sincerely apologize for not meeting the journal's formatting requirements. We fully understand the importance of proper formatting and acknowledge the areas where we did not comply with the guidelines.

To ensure the paper complies with the journal's formatting requirements, we have made comprehensive revisions. The specific measures taken are as follows:

1. **Reviewing the journal's formatting requirements**: We have carefully checked the journal's formatting guidelines and adjusted the paper's title, paragraphs, citation format, and layout of tables and figures to ensure that every element meets the journal's specific standards.
2. **Formatting and font specifications**: We have adjusted the line spacing and other formatting details to ensure the paper aligns with the journal's requirements, thus improving its overall readability.

We believe that these revisions have brought the paper in line with the journal's formatting standards and significantly enhanced its overall professionalism. Once again, we appreciate your valuable feedback on the paper, and we will complete these adjustments promptly, looking forward to your further review.

If you have any other suggestions during your review, please feel free to let us know. We will do our best to improve and meet all the journal's requirements.

***4.* Fig. 6** **have to be regenerated - Comparisons of the other model**

**Response:** Thank you for your feedback on the charts and figures in our paper, and for pointing out the need to regenerate the comparative images. We greatly value your comments and have recognized that the current images have some issues, such as insufficient clarity and unclear data presentation. To ensure the charts can more effectively showcase our research results, we have taken the following measures:

1. **Regenerating high-resolution comparative images**: We have ensured that the images now have sufficient resolution and detail to allow readers to accurately interpret the data. Additionally, we have adjusted the image size and format to meet the journal's requirements.
2. **Optimizing the presentation of comparisons**: When regenerating the comparative images, we have optimized the way the data is presented, using more intuitive colors, symbols, and legends to make the data comparison more apparent, thus avoiding potential misunderstandings.
3. **Enhancing figure explanations**: In addition to regenerating the images, we have added figure legends to ensure that the charts are fully explained.

We believe that the revised comparative images will now communicate our research findings more clearly. Once again, thank you for your valuable feedback. We have completed these revisions and submitted the new version for your review.

If you have any further suggestions or requests, we would be more than happy to continue making improvements.

**S6_Fig 6**

***5.* inside fig 7 the sub division of figures mentioned as 6a.,6b. same as Fig8. Clarity Needed.**

**Response:** Thank you for reviewing our paper and pointing out the errors in the figure captions. We highly value your feedback and sincerely apologize for any inconvenience this may have caused. After a careful review, we have indeed found that there were inaccuracies in the figure captions.

To address this issue, we have made the following corrections:

- **Correction of the captions:** We will thoroughly check all figure captions to ensure that each one is fully consistent with the content of the image, avoiding any misleading information.
- **Verification of consistency between charts and the main text:** We will also ensure that the captions of the figures align with the descriptions and analyses in the main text, so that readers can clearly understand the content of the figures.

We will update these captions in the revised version of the paper and ensure that there are no further errors in the figure captions. Thank you again for pointing out this issue; your feedback is extremely valuable in helping us improve the quality of our paper.

If you have any additional suggestions or areas that require further revision, we would be happy to make any necessary improvements. We look forward to your continued review.

**S7_Fig 7**

**S8_Fig 8**

**Thank you very much for your attention and time. Look forward to hearing from you.**

**Yours sincerely,**

**Miao Zhao**

**December 11, 2024**

**Shenyang University of Chemical Technology**
