# Supplementary material for: Left ventricular segmentation method based on optimized UNet and improved CBAM: ESV and EDV tracking study
Source: PLoS One. 2025 Jun 25;20(6):e0325794. doi: 10.1371/journal.pone.0325794 (PMC12193590; doi:10.1371/journal.pone.0325794)
Supplement: Attachment — Submitted filename: Response_to_Reviewers_auresp_2.docx [file pone.0325794.s005.docx]

**Dear reviewer,**

**We sincerely thank the editor and all reviewers for their valuable feedback that we have used to improve the quality of our manuscript. The reviewer comments are laid out below in italicized font and specific concern shave been numbered. Our response is given in normal font and changes/additions to the manuscript are given in Highlight Text.**

***Reviewer 1***

***1.Words are frequently hyphenated at the end of lines, with the second part appearing at the beginning of the next line. This occurs throughout the article.***

**Response:** Thank you for your careful review and your suggestion regarding hyphenation at line breaks. I have addressed the hyphenation issues caused by line breaks as you suggested. All related instances have been resolved in the revised manuscript.

We have comprehensively revised the manuscript based on your feedback and ensured that these issues have been properly addressed. Once again, thank you for your careful review of our paper and your valuable suggestions. We have completed these revisions and submitted the revised version, looking forward to your further review.

If you have any other suggestions or requests, we are very happy to continue making improvements. Relevant figures/screenshots have also been attached for your reference.

***2.Table 4 uses a different font.***

**Response:** Thank you very much for kindly pointing out the font inconsistency in Table 4. I sincerely appreciate your meticulous attention to such important details, which has helped us further improve the quality of our manuscript. In accordance with your suggestion, we have carefully revised Table 4 and ensured that the font has been unified to Times New Roman, consistent with the rest of the manuscript. We have also double-checked all tables and figures to avoid similar issues elsewhere.

We truly value your constructive feedback and professional guidance. Your careful review and valuable comments have played a crucial role in enhancing the overall quality and presentation of our work. If you have any other suggestions or requests, we are more than happy to make further improvements as needed. Relevant figures and screenshots have also been attached for your reference.

Once again, thank you very much for your support and assistance throughout the review process. We greatly appreciate your time and effort, and we look forward to your further comments and guidance.

**Table 4**

***3.*** ***The legend of Figure 6 uses a different font.***

**Response:** Thank you very much for kindly pointing out the font inconsistency in the legend of Figure 6. I sincerely appreciate your careful review and your keen attention to detail, which has greatly helped us improve the overall quality and professionalism of our manuscript. Following your valuable suggestion, I have carefully revised the legend of Figure 6, ensuring that the font is now fully consistent with the rest of the manuscript, using Times New Roman throughout for uniformity. Additionally, I have double-checked all other figures and legends to make sure there are no similar issues elsewhere.

Your constructive feedback and rigorous standards are of great significance to us. We truly value your time, effort, and professional input throughout the review process. If you have any further comments, suggestions, or specific requirements, please do not hesitate to let us know. We are always willing and eager to make additional improvements to meet the journal’s high standards and your expectations.

Relevant figures and screenshots have also been attached for your reference. Once again, thank you for your valuable guidance and continuous support. We look forward to your further comments and hope that our revisions will meet with your approval.

**Fig 6**

***4.*** ***The article lacks proper alignment.***

**Response:** Thank you very much for pointing out the issue of improper alignment in the article, and for your meticulous attention to formatting details. I have carefully revised the manuscript and applied justified alignment throughout to ensure that the formatting is consistent and properly aligned across the entire document.

Your thoughtful feedback and high standards are invaluable to us, as they help us continuously improve the quality and professionalism of our work. If you have any further suggestions, requests, or specific requirements, please feel free to let us know at any time. We greatly appreciate your time and effort in reviewing our manuscript, and we are always eager to make further improvements based on your guidance.

Once again, thank you for your kind support and constructive comments. We look forward to your further suggestions and sincerely hope our revisions will meet your expectations.

***5. The right side of Figure 1 (the yellow block diagram) is not clear.***

**Response:** Thank you very much for kindly pointing out that the right side of Figure 1 (the yellow block diagram) was not clear. I sincerely appreciate your careful review and your attention to such important details, which have greatly contributed to improving the overall quality of our manuscript. In response to your suggestion, I have revised Figure 1 by changing the background of the right block diagram to light blue and increasing the font size, which has significantly enhanced its clarity and readability.

Your constructive feedback and rigorous standards are of great value to us, and we are grateful for your time and effort in reviewing our work. If you have any further comments, suggestions, or specific requests, please feel free to let us know at any time. We are always willing to make additional improvements to ensure the highest standards of quality.

Relevant figures and screenshots have also been attached for your reference. Once again, thank you for your valuable guidance and continued support. We look forward to your further comments and sincerely hope that our revisions will meet your expectations.

**Fig 1**

***6.The reference section does not provide complete author lists. "Et al." is used in many places, the full list should be provided.***

**Response:**Thank you very much for pointing out that the reference section did not provide complete author lists and that "et al." was used in many places. I sincerely appreciate your careful review and your attention to detail, which has greatly contributed to improving the academic rigor and completeness of our manuscript. In accordance with your suggestion, I have carefully revised all references and included the full list of authors for each entry to ensure accurate and comprehensive attribution.

Your constructive feedback and high standards are extremely valuable to us, and we are grateful for the time and effort you have devoted to reviewing our work. If you have any further suggestions or requests, please feel free to let us know at any time. We are always happy and willing to make additional improvements based on your guidance.

Relevant figures and screenshots have also been attached for your reference. Once again, thank you for your valuable input and continued support. We look forward to your further comments and sincerely hope our revisions meet your expectations.

***Reviewer 3***

***1.*** ***The reported 1.05% improvement in Dice is modest—statistical significance tests (e.g., p-values or confidence intervals) would strengthen this.***

**Response:** Thank you very much for your valuable suggestion regarding the statistical significance of the reported 1.05% improvement in the Dice coefficient. We sincerely appreciate your careful review and your focus on the scientific rigor of our study. In response to your advice, we have included statistical significance tests in our experiments and have added confidence interval information in the manuscript to further strengthen the credibility and robustness of our results.

Your constructive feedback and high standards have greatly contributed to improving the overall quality and reliability of our work. If you have any other suggestions, comments, or specific requests, please feel free to let us know. We are always eager to continue improving our manuscript based on your guidance.

Relevant figures and screenshots have also been attached for your reference. Once again, thank you for your valuable input and continuous support. We look forward to your further comments and sincerely hope our revisions will meet your expectations.

**P 15**

***2.Visual comparisons (e.g., segmentation overlays) were promised but could be more prominently and consistently used.***

**Response:** Thank you very much for your valuable suggestion regarding the use of visual comparisons, such as segmentation overlays. We sincerely appreciate your careful review and insightful feedback, which have played an important role in enhancing the clarity and presentation quality of our manuscript. In response to your feedback, we have revised our visualization approach: the ground truth annotations are now displayed in white, while the segmentation results from different models are shown in red. This not only makes the visual comparisons more prominent, but also ensures greater consistency and clarity in our presentation.

Your constructive suggestions and high standards are greatly appreciated and have been instrumental in improving our work. If you have any other suggestions, comments, or specific requests, please feel free to let us know at any time. We are always willing to make further improvements to meet the highest standards.

Relevant figures and screenshots have also been attached for your reference. Once again, thank you for your valuable guidance and continuous support. We look forward to your further comments and sincerely hope our revisions will meet your expectations.

**Fig 7**

**Fig 8**

***3.Inference speed or runtime performance is not clearly benchmarked—important for real-time clinical utility claims***

**Response:** Thank you very much for highlighting the importance of evaluating inference speed and runtime performance for real-world clinical applications. We sincerely appreciate your careful review and your focus on the practical value of our research. In response to your suggestion, we have measured the computation time per batch for different models, calculated their inference speeds (inferences per second), and compared their computational complexity and parameter counts. These comprehensive runtime performance evaluation results have been added and reported in the revised manuscript to provide a clearer understanding of the models’ suitability for real-time clinical use.

Your constructive feedback and high standards have been instrumental in helping us improve both the quality and the clinical relevance of our work. If you have any other suggestions, comments, or specific requests, please feel free to let us know at any time. We are always happy to make further improvements based on your valuable guidance.

Relevant figures and screenshots have also been attached for your reference. Once again, thank you for your valuable input and continued support. We look forward to your further comments and sincerely hope that our revisions will meet your expectations.

**P 15**

***4.Overuse of passive voice and vague terms (e.g., “it is proposed that…”).***

**Response:** Thank you very much for pointing out the overuse of passive voice and vague terms (e.g., “it is proposed that…”) in the manuscript. We sincerely appreciate your careful review and insightful feedback, which have been invaluable in helping us improve the overall quality of our writing. In response to your suggestion, we have carefully revised the text to use more explicit and active voice expressions, thereby improving the clarity, readability, and precision of our manuscript.

Your constructive comments and high standards have greatly contributed to enhancing both the scientific rigor and the overall presentation of our work. If you have any further suggestions, comments, or specific requests, please do not hesitate to let us know. We are always happy to make further improvements to ensure the highest quality of our manuscript.

Once again, thank you for your valuable guidance and continuous support. We look forward to your further comments and sincerely hope that our revisions will meet your expectations.

***5.Some paragraphs are overly long and would benefit from clearer topic sentences and transitions.***

**Response:** Thank you very much for pointing out that some paragraphs were overly long and would benefit from clearer topic sentences and transitions. We sincerely appreciate your careful review and insightful suggestions, which have been instrumental in improving the overall structure and readability of our manuscript. In response to your advice, we have carefully split the lengthy paragraphs and added more explicit topic sentences and transitional phrases to enhance the clarity, coherence, and logical flow of the manuscript.

Your constructive feedback and high standards are invaluable to us, and we are deeply grateful for the time and effort you have dedicated to reviewing our work. If you have any further suggestions, comments, or specific requests, please feel free to let us know at any time. We are always happy to continue making improvements based on your valuable guidance.

Once again, thank you for your support and thoughtful comments. We look forward to your further input and sincerely hope our revisions will meet your expectations.

***6.*** ***Inconsistent spacing, alignment, and formatting across sections.***

**Response:** Thank you very much for pointing out the inconsistent spacing, alignment, and formatting across different sections. We sincerely appreciate your careful review and attention to such important details, which are essential for ensuring the professionalism and readability of our manuscript. In response to your suggestion, we have carefully reviewed the entire manuscript and thoroughly standardized the spacing, alignment, and formatting in all sections to ensure a consistent and polished presentation.

Your constructive feedback and high standards have been invaluable in helping us further improve the quality of our work. If you have any additional suggestions, comments, or specific requests, please do not hesitate to let us know. We are always happy to continue making improvements and refinements based on your valuable guidance.

Once again, thank you for your support and thoughtful input. We look forward to your further comments and sincerely hope that our revisions will meet your expectations.

***7.*** ***Some promises made in the response (e.g., simplified figures, added analogies) should be double-checked in the revised manuscript to ensure they were implemented.***

**Response:** Thank you very much for reminding us to double-check the implementation of the promised changes in our response (such as simplified figures and added analogies). We have carefully reviewed the revised manuscript to ensure that all these promised modifications have been incorporated. If there are any remaining issues, please let us know, and we will address them promptly.

**1.Add Analogies**:In our previous response, we clarified some abstract or complex concepts related to CBAM and SimAM by using real-world analogies, and these have already been included in the manuscript. When explaining CBAM, we compared it to observing a busy street filled with people, vehicles, and buildings. If you are looking for a friend, your brain naturally focuses on the crowd, ignoring irrelevant elements like parked cars or buildings. For SimAM, we used the analogy of a classroom where a teacher is explaining a topic. Some students (features) are more attentive, having learned more and gained a deeper understanding of the material. SimAM acts like a teacher who can identify which students are more focused and direct more attention to them, thereby improving the overall learning outcome. It pays more attention to those students who have a deeper understanding (important features), while ignoring those who are less engaged (irrelevant features).

**P 8**

**P 10**

***2.Simplified figures***: In our previous response, we redrew S6_Fig 6 to ensure that the image had sufficient resolution and detail, allowing readers to accurately interpret the data. At the same time, we adjusted the image size and format to meet the journal’s requirements. To optimize the comparison, we used more intuitive colors, symbols, and legends in the newly generated image, making the differences between the data clearer and reducing potential misunderstandings. In addition, we supplemented and improved the legend explanations to ensure that all chart content is fully explained. In this revision, we also redrew S7_Fig 7 and S8_Fig 8 to make the visual comparisons more prominent and easier to understand.

If you have any other suggestions or requests, we are very happy to continue making improvements. Relevant figures/screenshots have also been attached for your reference.

**Fig 6**

**Fig 7**

**Fig 8**

***8.*** ***Briefly mention future directions (e.g., generalizability testing on MRI or CT, or deployment in edge devices).***

**Response:** Thank you very much for your valuable suggestion. We have added a brief discussion of possible future directions, such as generalizability testing on MRI or CT data and the deployment of the model in edge devices. Your feedback has been very helpful in enhancing the depth and breadth of our manuscript, and we sincerely appreciate your thoughtful input.

Your constructive comments and high standards have played a crucial role in improving the overall quality and scientific value of our work. If you have any further suggestions, comments, or specific requests, please feel free to let us know at any time. We are always happy to continue refining and enhancing our manuscript based on your guidance.

Relevant figures and screenshots have also been attached for your reference. Once again, thank you for your valuable input and ongoing support. We look forward to your further comments and sincerely hope that our revisions will meet your expectations.

**P 17**

**Thank you very much for your attention and time. Look forward to hearing from you.**

**Yours sincerely,**

**Miao Zhao**

**May 17, 2025**

**Shenyang University of Chemical Technology**
